# Optimized ultrasonic‐assisted extraction of papaya seed oil from Hainan/Eksotika variety

**DOI:** 10.1002/fsn3.1125

**Published:** 2019-07-08

**Authors:** Weimin Zhang, Yong‐Gui Pan, Wuyang Huang, Haiming Chen, Hong Yang

**Affiliations:** ^1^ College of Food Science & Technology Huazhong Agricultural University Hubei China; ^2^ College of Food Science Hainan University Hainan China; ^3^ Institute of Farm Product Processing & Jiangsu Key Laboratory for Horticultural Crop Genetic Improvement Jiangsu Academy of Agricultural Sciences Jiangsu China

**Keywords:** oxidative stability, papaya (*Carica papaya* Linn) seed oil, response surface methodology, ultrasound‐assisted extraction

## Abstract

Hainan/Eksotika papaya is a popular cultivated plant in Hainan Island, China. Papaya seed oil (PSO) contains functional compounds with good antioxidant activity, especially monounsaturated fatty acids. In this work, the ultrasound‐assisted extraction (UAE) of PSO was optimized using response surface methodology. It was found that the optimal extraction performance was realized when the elevated time was set to 20 min, the ultrasound power was set to 250 W, and the n‐hexane‐to‐sample ratio was set to 16:1 (v/w). The highest yield of PSO (32.27%) was obtained under the optimal conditions, and PSO showed good oxidative stability. Differential scanning calorimetry analysis showed that the melting point of Hainan/Eksotika PSO was low, while its crystallization temperature was high. FTIR and NMR were used to analyze the chemical structure of PSO, which also proved that PSO possessed good stability without oxidative degradation. In addition, scanning electron micrograph was employed to investigate the change in seed microscopic structure. The results showed UAE caused serious structural damage of sample cell membranes and walls, which help oil access to the solvent with a high extraction ratio. The results indicated that UAE is an efficient environmental‐friendly, and promising technique could be applied to produce PSO or other edible oil with a better health‐beneficial value in food industry.

## 1 INTRODUCTION

Papaya is mainly used for processing candy, jam, jelly, and pickles (Chávez‐Quintal, González‐Flores, Rodríguez‐Buenfil, & Gallegos‐Tintoré, 2011). The seeds in papaya fruits, accounting for nearly 20% of its total weight (Chielle, Bertuol, Meili, Tanabe, & Dotto, 2016), can be potentially valuable because of nutritional and functional constituents contained. In addition, the seed of papaya is edible and can be taken as a substitute of black pepper for its spicy flavor. In fact, papaya seed contains 27.3%–28.3% protein, 28.2%–30.7% lipids, and 19.1%–22.6% crude fibers; however, it has not been commercially utilized. Most of the seeds are generated in the form of dregs and discarded as an agricultural waste during the fruit processing, causing environmental problems. To make a zero‐waste strategy supporting the green technology in a more ecologic, economic, and innovative way, systematic studies on such a valuable waste are needed. Extracting oil from papaya seeds was a successful attempt. Papaya seeds had a high content of oil (13.9%–30.7%), which was full of monounsaturated fatty acids and functional phytochemicals, such as tocopherol, carotenoid, and phenolics. Moreover, papaya seed oil (PSO) was found to be robust against oxidation, which can be processed into a new type of cooking oil containing a better health‐beneficial value in food industry (Samaram, Mirhosseini, Tan, & Ghazali, 2014). This provides insight into reducing environmental pollution and making waste seeds profitable. Therefore, more and more attentions have been diverted to the PSO.

Normally, the output and quality of extracted PSO are significantly associated with the extraction method and extraction condition. Soxhlet solvent extraction is a common method to extract oil from plant sources, but the process of this method is relatively time consuming and the recovery of oil is limited. Therefore, many efforts have been made to develop methods integrating thermal and mechanical treatments to enhance PSO extraction. Among them, ultrasound‐assisted extraction (UAE) has been proved to be an efficient and economic “green” technology for the extraction of oil, with high oil yields, short extraction time, and a low volume of solvent (Tavares, Massa, Gonçalves, Silva, & Santos, 2017). In addition, UAE makes it possible to extract a high rate of bioactive compounds at relatively lower temperature, resulting in higher quantity and quality of final products than Soxhlet extraction (SE; Miklavcic, 2017).

The effects of temperature, time, solvent–solid ratio, ultrasound power on the extraction of oil from Batek Batu, and Sekaki were investigated. Most studies simply evaluated the yield and chemical composition of PSO treated with ultrasound, and very few studies focused on the effects of UAE on physicochemical characteristics of PSO. Especially, there is no report on the changes in seed microscopic structures before and after UAE extraction. In this study, Soxhlet and UAE were used to extract PSO from Hainan/Eksotika papaya, and the two extraction methods were compared. To optimize the UAE of PSO from Hainan/Eksotika variety, response surface optimization was adopted using n‐hexane as a solvent. This study investigated the yield and physicochemical characteristics of PSO extracted from Hainan/Eksotika variety by UAE. Furthermore, the mechanism of UAE was investigated using scanning electron microscopy (SEM). This study provides an environmentally friendly extraction method and develops a new edible oil resource, which has positive effects on the food industry.

## 2 MATERIALS AND METHODS

### 2.1 Plant materials and chemical reagents

Hainan/Eksotika papaya was purchased from a local market in Haikou, Hainan, China. Seeds of papaya fruit were collected, washed and dried at 60°C for 2 days, and finally ground into powder and sieved via a 40‐mesh screen (Samaram et al., 2015). Standards including nonanal. octanal, 2‐decenal, decanal, and 2‐undecenal were purchased from Tokyo Kasei Kogyo Co., Ltd. Isopropanol and acetonitrile for HPLC were chromatographically pure. All the other chemicals and solvents were of analytical grade.

### 2.2 Soxhlet extraction

Seed powder (5 g) was extracted and refluxed using a soxhlet extractor with 200 ml of n‐hexane (60°C) for 10 hr at a mass‐to‐solvent ratio of 1:40 (w/v), resulting in seed oil according to Bhutada, Jadhav, Pinjari, Nemade, and Jain (2016) with some modification. The resulted seed oil was collected by rotary vacuum evaporator at 60°C and then stored at 4°C.

The extraction yield can be obtained by the following equation:

(1) Extraction yield(%)=extracted oil(g)/initial seed power(g)×100.

### 2.3 Experimental design of UAE

Before entering overall implementation of Box‐Behnken central composite design, single‐factor experiments were carried out in an indirect ultrasound bath. The variables evaluated in this study were as follows: solvent (acetone, ethyl acetate, n‐hexane, petroleum ether), ultrasound power (150–250 W), time (5–50 min), and solvent–sample ratio (6:1–16:1, v/w), and these variables were investigated.

The effects of ultrasound power, extraction time, and solvent–sample ratio on the PSO yield were, respectively, analyzed using RSM. The single‐factor experiment was carried out to determine the contribution of each variable, as shown in Table 1. The optimal conditions were obtained using Box–Behnken Design (BBD). The average yield (%) of oil was regarded as response value. Table 1 shows operating parameters suggested by SAS 8.0 (Statistical Analysis System Institute Inc.). According to the following quadratic polynomial regression equation, predicted yield of PSO was obtained.

(2) $$Y=\beta_{0}+\sum_{i=1}^{3}\beta_{i}X_{i}+\sum_{i=1}^{3}\beta_{\mathrm{ii}}X_{i}^{2}+\sum_{i=1}^{2}\sum_{j=i+1}^{3}\beta_{\mathrm{ij}}X_{i}X_{j},$$

where *Y* represents the predicted response; *β*_0_, *β*_i_, *β*_ii_, and *β*_ij_ are regression coefficients of the intercept, linear, quadratic, and interaction terms, respectively; and *X*_i_ and *X*_j_ are independent variables (Wang, Wang, Wang, Xiao, & Liu, 2016).

**Table 1:** Box–Behnken design of process variables along with experimental values for the yield response

No.	*X*_1_ Ultrasonic power (W)	*X*_2_ Ultrasonic time (min)	*X*_3_ Solvent‐to‐sample ratio (g/ml)	Extraction yield (%)
1	−1 (200)	−1 (15)	0 (14:1)	24.06
2	−1	1 (25)	0	25.25
3	1 (250)	−1	0	27.90
4	1	1	0	29.39
5	0 (225)	−1	−1 (12:1)	26.44
6	0	−1	1 (16:1)	29.93
7	0	1	−1	25.87
8	0	1	1	28.98
9	−1	0 (20)	−1	24.28
10	1	0	−1	31.92
11	−1	0	1	26.77
12	1	0	1	32.40
13	0	0	0	24.69
14	0	0	0	25.15
15	0	0	0	25.58

### 2.4 Physicochemical characteristics of PSO

The acid value (AV), free fatty acid contents, iodine value, *p*‐anisidine value (PAV), peroxide value (PV), saponification value, and specific gravity of PSO were evaluated according to the methods prescribed by American Oil Chemists Society. The lovibond tintometer (Shanghai Technologies) was used to measure the color of oil. A hand‐held refractometer was used to test the refractive index. The oxidative stability of PSO was evaluated using PV and PAV. Totox value (TV) can be calculated by the following formula (Samaram et al., 2014):

(3) TV=2PV+PAV.

### 2.5 DSC‐based thermal analysis

A Perkin‐Elmer DSC with a data station was used for thermal analysis. The purge gas was nitrogen (99.99% purity). Melted sample (3 g) was added in a DSC pan and then hermetically sealed (Yanty, Marikkar, Nusantoro, Long, & Ghazali, 2014). In contrast, a blank control was put in an empty sealed DSC pan. The oil sample was placed at 60°C for 2 min, then cooled to −60°C by 5°C/min, let stand for 2 min, and finally heated to 60°C by 5°C/min (Da Silva, Arellano, & Martini, 2019; Tan, Chong, Hamzah, & Ghazali, 2018). The activation energies (*E*_a_, kJ/mol) and frequency factors (*A*, per hour) for lipid oxidation in oils were calculated using the Arrhenius equation (Equation 4).

(4) $$\ln\left(k\right)=\ln\left(A\right)-\left(E_{\text{a}}/\text{RT}\right),$$

where *k* is the kinetic rate constant (per hour), and *R* is the molar gas constant (8.314 J/mol K).

### 2.6 FTIR analysis of PSO

The FTIR spectra of PSO were recorded using a FTIR spectrophotometer (Nicolet 6700; Thermo scientific) to reveal the functional groups by recording 45 scans in transmittance mode in the range of 4,000–400/cm (Bhutada et al., 2016).

### 2.7 Proton nuclear magnetic resonance (^1^HNMR) analysis of PSO

The molecular structure of the PSO extracted by UAE was analyzed using ^1^HNMR. The oil sample was dissolved in the deuterated chloroform. ^1^HNMR spectra of the extracted PSO were recorded using a Bruker DRX‐400 NMR spectrometer (Bruker Instruments) with ^1^H nuclei observed at 400 MHz (Almoselhy, Allam, El‐Kalyoubi, & El‐Sharkawy, 2014).

### 2.8 SEM analysis

The morphological changes in seed samples before and after UAE were determined using a Quanta‐200 environmental scanning electron microscope system (FEI Company). Samples were fixed and then sputtered with a thin layer of gold. Determination was operated at an accelerating voltage of 12.5 kV under high vacuum condition (1,000 × magnification) (Jiao et al., 2014).

### 2.9 Aldehyde composition analysis of PSO using HPLC

Cao et al. (2014) conducted HPLC to measure the composition and content of volatile aldehyde in PSO extracted by UAE and SE, respectively. The oil sample (100 mg) was dissolved into isopropanol (100 ml). Then, 1 ml of the sample solution was added to 2,4‐dinitrophenol solution, resulting in the mixed solution. After that, the mixed solution was heated at 45°C for 30 min, cooled to room temperature, and then filtered for HPLC analysis. Using octanal, nonanal, 2‐decenal, decanal, and 2‐undecenal as standards, HPLC analysis was carried out on a Shimadzu HPLC system. The acetonitrile/H_2_O solution (80:20, v/v) was used as elution solution, of which the flow rate was set to 1.3 ml/min and the injection volume was set to 20 μl.

### 2.10 Statistical analysis

All experiments were replicated three times. Data were expressed in the form of means ± standard deviation (*SD*). The data were analyzed by the IBM SPSS statistical software (SPSS Inc.). The difference was of statistical significance at *p* < 0.05.

## 3 RESULTS AND DISCUSSION

### 3.1 Optimization of UAE process

The results of single‐factor experiments indicated that the extraction yield of PSO was associated with by solvent, ultrasound power, extraction time, and solvent–sample ratio. The highest yield was obtained when using n‐hexane as extract solvent. The maximal UAE yield of PSO could be obtained by optimizing the conditions. Table S1 and Figure S1 showed that the regression model was precise. There were significant differences (*p* < 0.05 or *p* < 0.01) in the linear terms (*X*_1_ and *X*_3_) and a quadratic term (*X*_3_^2^), while there were no significant differences (*p* > 0.05) in one linear term (*X*_2_), three interaction terms (*X*_1_ *X*_2_, *X*_1_ *X*_3_, and *X*_2_ *X*_3_), and two quadratic terms ($X_{1}^{2}\;\text{and}\;X_{2}^{2}$). The oil yield calculated can be expressed using the second order polynomial model:

$$\begin{array}{c} Y=25.14+2.65625X_{1}+0.145X_{2}+1.19625X_{3}\\ +1.27375X_{1}^{2}+0.075X_{1}X_{2}-0.5025X_{1}X_{3}\\ +0.23625X_{2}^{2}-0.095X_{2}X_{3}+2.42875X_{3}^{2}, \end{array}$$

where *Y* represents the yield (%), *X*_1_ stands for the ultrasonic power (W), *X*_2_ represents the extraction time (min), and *X*_3_ represents the solvent–sample ratio.

The influences of three extraction parameters can be ranked as follows: ultrasonic power > solvent–sample ratio > ultrasonic time. The ultrasonic power showed the greatest influence on the PSO yield in the UAE, and this effect was clearly observed when comparing the results of experiments 1 and 3 in Table 1. Tavares et al. (2017) found that an increase in the variables time, temperature, and solvent: seed ratio favored the removal of oil from the crambe seeds. In addition, significant higher PSO yields were obtained when the solvent‐to‐solid ratio increased, due to a higher driving force and a less viscosity in the more diluted solution (de Mello, dos Santos Garcia, & da Silva, 2017). A literature reported by Stevanato and da Silva (2019) evaluated the extraction of radish seed oil at 45°C and 60 min and observed that increasing the solvent‐to‐seed ratio from 4:1 to 12:1 increased the oil yield from 17.10% to 23.22%, respectively. Another study reported an increase in the yield of macauba pulp oil (28.15%–30.70%) when increasing the relative amount of solvent from 6:1 to 10:1 (Rodrigues, Mello, dos Santos Garcia, & Silva, 2017). Extraction time is also an important variable which has been found to have the greatest influence on the radish seed oil (*Raphubus sativus* L.) yield (da Silva, dos Santos Garcia, Arroyo, & da Silva, 2017). In this study, extraction yield of PSO increased from 27.90% to 29.39% when ultrasonic time increased from 15 to 25 min under ultrasonic power of 250 W and solvent‐to‐sample ratio of 14:1. The result may be due to the increased rupture of cell walls during a longer sonication time, improving the mass transfer of intracellular products to the solvent (Samaram et al., 2015).

The optimal extraction performance could be obtained when ultrasonic power was 249.59 W, extraction time was 20.34 min, and solvent–sample ratio was set to 16.22:1 (v/w) (Fig. S1), which is in well consistence with the results of others using the response surface (Jadhav, Holkar, Goswami, Pandit, & Pinjari, 2016; Li, Lu, et al., 2012; Li, Qu, Zhang, & Wang, 2012). In view of the actual operation, ultrasonic power was set to 250 W, extraction time was set to 20 min, and solvent–sample ratio was set to 16:1, respectively. The actual PSO yield obtained under optimum conditions was 32.27 ± 0.39%, which was very close to the predicted value of 32.55% of the regression model. This suggests that the response model could predict and evaluate the oil yields from papaya seed.

### 3.2 Extraction yield of PSO

Table 2 shows the yield and physicochemical properties of PSO extracted by SE and UAE method, respectively. It could be observed that the yield of PSO using SE was only 25.27%, which was lower than that using UAE (32.27%). Similar results were reported from other varieties. By using SE to extract oil for several hours, 30.7% PSO was obtained from Malaysia/Batek Batu, while 28.3% was from Kaeg‐dum and Hawaii, 29.16% from Brazil/Formosa, and 27.0% from Hong Kong/Sekaki (Zhang et al., 2018). Therefore, UAE produced a high yield and required much shorter time (20 min) than SE method (8–10 hr).

**Table 2:** Comparison of physicochemical properties of papaya seed oil in different extraction methods

Physicochemical properties	Obtained values		FAO/WHO standard	Literature values
	Soxhlet extraction	Ultrasound‐assisted extraction		
Color (Lovibond, 1 in.)	46Y, 4.5R	32Y, 5.5R	–	
Relative density (g/cm^3^)	0.84 ± 0.01a	0.87 ± 0.02a	–	
Refractive index (30°C)	1.4624 ± 0.0007a	1.4672 ± 0.0003a	–	1.4581–1.4678
Iodine value (g I_2_ /100 g)	64.30 ± 1.49a	61.73 ± 1.31a	80–106	64.1–79.95
Saponification value (mg KOH/g)	153.96 ± 8.97b	209.07 ± 4.86a	181.40	96.4–197
Acid value (mg KOH/g)	1.61 ± 0.03a	1.26 ± 0.05b	4	
Peroxide value (meq/kg)	1.35 ± 0.09a	1.03 ± 0.02b	–	
*p*‐anisidine value (meq/kg)	5.40 ± 0.60a	3.43 ± 0.25b	–	
Totox value (meq/kg)	8.10 ± 0.26a	5.49 ± 0.29b	–	
Free fatty acid (%)	0.81 ± 0.02a	0.63 ± 0.03b	0.57–0.728	0.33–1.311
Yield (%)	25.27 ± 0.88b	32.27 ± 0.39a		

Note

Mean ± *SD*, *n* = 3. Different letters are significantly different among samples (*p* < 0.05) according to Fisher's least significant difference procedure.

### 3.3 Physicochemical properties and oxidative stability of PSO

As shown in Table 2, the PSO extracted from Hainan Eksotika using UAE had smaller color values (32Y, 5.5R) than that using SE (46Y, 4.5R), which is consistent with the study results by Samaram et al. (2014). UAE might obtain purer PSO with little pigments than SE. Previous study also reported that the oil color from Malaysia Sekaki and Hong Kong/ Sekaki was golden yellow, while the oil color from Batek Batu was reddish yellow. Generally, the difference in color value of PSO extracted from Hainan Eksotika was due to the difference in papaya varieties and/or extraction methods.

Relative density of UAE and SE was 0.87 ± 0.02 and 0.84 ± 0.01 g/cm^3^, respectively. In addition, the refractive index was 1.4624 ± 0.0007 with SE and 1.4672 ± 0.0003 with UAE, which is larger than that (1.4581) reported by Marfo, Oke, and Afolabi (1986) and consistent to that reported by Malacrida, Kimura, and Jorge (2011).

The iodine value was 64.30 ± 1.49 g I_2_/100 g for PSO extracted by SE, while it was 61.73 ± 1.31 g I_2_/100 g for PSO extracted UAE. Hainan Eksotika PSO had a lower iodine value than other varieties, such as Malaysia/Batek Batu (66.0–69.3 g I_2_/100 g), Kaeg‐dum and Hawaii (72.5–74.9 g I_2_/100 g), Taiwan/Tainoung (64.1 g I_2_/100 g), Brazil/Formosa (79.95 g I_2_/100 g), Hong Kong/Sekaki (76.90 g I_2_/100 g), and Malaysia Sekaki (71.00–71.50 g I_2_/100 g). This further confirms its nondrying oil properties. The saponification value (209.07 ± 4.86 mg KOH/g) of Hainan Eksotika PSO extracted by UAE was much higher than Hainan Eksotika PSO extracted by SE (153.96 ± 8.97 mg KOH/g) and other varieties (96.4 – 197.0 mg KOH/g) (Tekin, Akalın, & Şeker, 2015).

It was found that the specific gravity, refractive index, PV, AV, iodine value, TV, saponification value, and the content of free fatty acid of the oil extracted by UAE were significantly lower than those extracted by SE (*p* < 0.05). However, the extraction methods did not make significant impact (*p* > 0.05) on the fatty acid composition of PSO, or on the fatty acid compositions of sour cherry kernel oil (Samaram et al., 2014) and PSO (Phan, Junyusen, Liplap, & Junyusen, 2018).

As shown in Table 3, the oxidative stability was greatly (*p* < 0.05) influenced by the extraction methods. The AV of UAE (1.26 ± 0.05 mg KOH/g) was significantly (*p* < 0.05) lower than that of SE (1.61 ± 0.03 mg KOH/g). In addition, PV, PAV, and TV in UAE (1.03 ± 0.02, 3.43 ± 0.25, and 5.49 ± 0.29 meq/kg, respectively) were significantly (*p* < 0.05) lower than those in SE (1.35 ± 0.09, 5.40 ± 0.60, and 8.10 ± 0.26 meq/kg, respectively). The results may be due to the mild UAE conditions (lower temperature, a shorter period of time) which is benefit for retaining more antioxidants, such as polyphenols, tannins, and anthocyanins (Da Porto, Porretto, & Decorti, 2013). Therefore, the oxidative stability of UAE‐PSO was stronger than that of SE‐PSO.

**Table 3:** Calculation of activation energy (*E*a) of papaya seed oil obtained using ultrasound‐assisted extraction (UAE) and Soxhlet extraction (SE) using the Kissinger–Akahira–Sunose method

Heating rate β (°C/min)	SE				UAE			
	*lg*（β/T^2^）	*T*_p1_ (°C)	*T*_p1_ (K)	*k*	*lg*（β/T^2^）	*T*_p1_ (°C)	*T*_p1_ (K)	*k*
5	−4.94	389.67	662.82	−4.97	402.83	675.98	−4.391	−4.94
10	−4.66	403.17	676.32	−4.97	421.33	694.48	−4.391	−4.66
15	−4.51	423.50	696.65	−4.97	440.50	713.65	−4.391	−4.51
20	−4.40	438.33	711.48	−4.97	449.33	722.48	−4.391	−4.40
linear equation	*y* = −4.9721*x* + 2.6163				*y* = −5.5106*x* + 3.2102			
*R* ^2^	0.9387				0.9843			
*E*_a_ (kJ/mol)	90.51				100.32			

### 3.4 Thermal behavior

**Figure 1:**
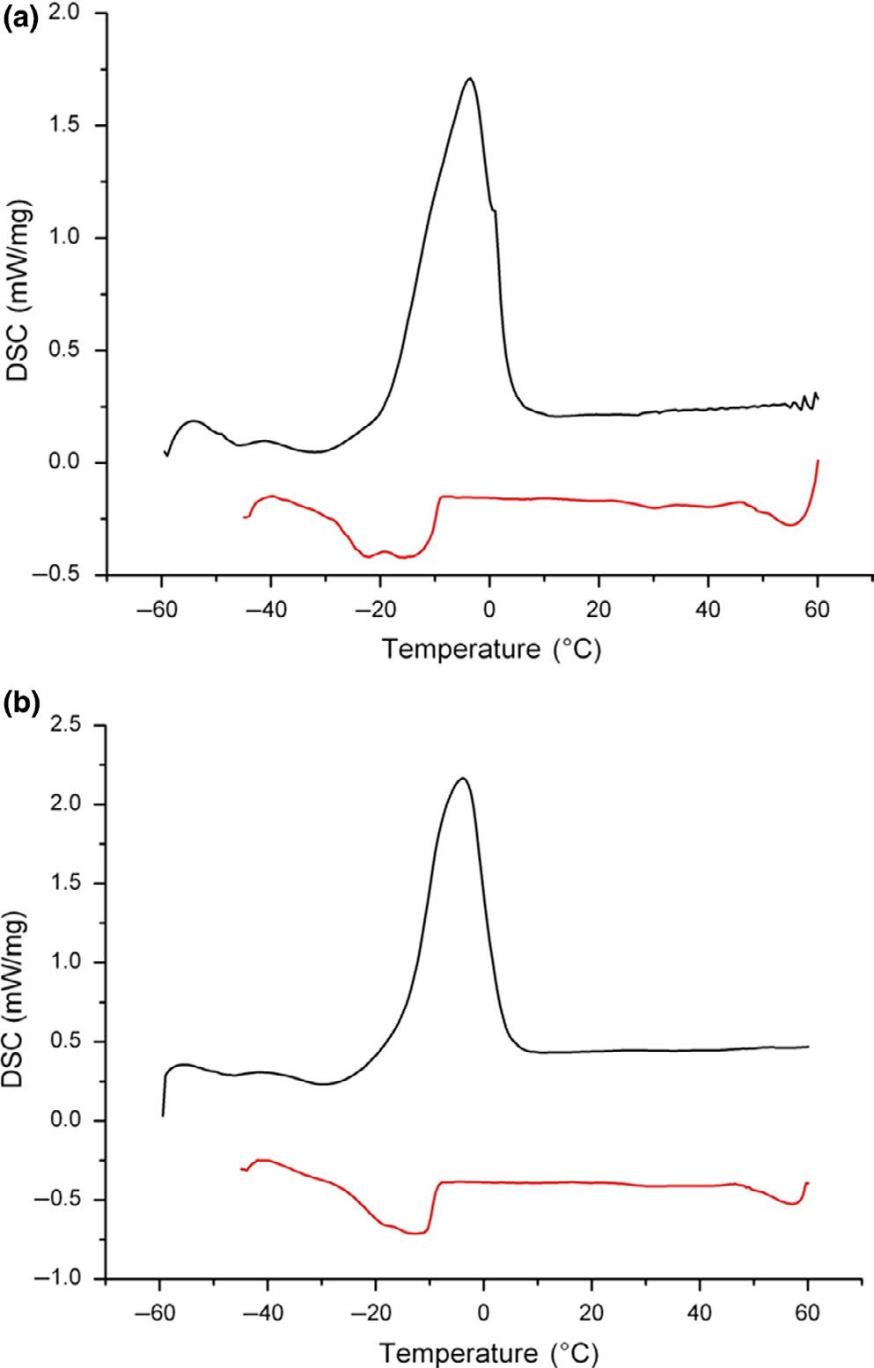
DSC spectra of papaya seed oil (PSO) from (a) Soxhlet extraction (n‐hexane, 60°C, 10 hr, 1:40 [w/v]), and (b) ultrasound‐assisted extraction (n‐hexane, 60°C, 20 min, 1:16 [w/v], 250 W). Black line: thermal behavior; red line: crystallization behavior

Figure 1 shows the thermal and crystallization behaviors of SE‐PSO and UAE‐PSO. From the melting curve (A) of SE‐PSO, we can observe a broad endothermic heat transition at 0.9°C, with a shoulder peak at −3.6°C. From the crystallization curve (B), we can see two exothermic heat transitions, with smaller peaks at −22.1°C and −15.6°C, respectively. For UAE‐PSO, it could be seen a sharp narrow peak at −3.9°C in the melting curve and a smaller broad peak at −12.5°C in the crystallization curve. In this study, Hainan Eksotika PSO had a lower melting point (−3.6°C to 0.9°C for SE‐PSO, −3.9°C for UAE‐PSO) and a higher crystallization temperature point (−22.1°C to −15.6°C for SE‐PSO, −12.5°C for UAE‐PSO). The melting point and crystallization temperature were 10.5°C and −42.2°C for PSO extracted from Batek Batu and 12.4°C and −48.2°C for that extracted from Hong Kong/Sekaki (Yanty et al., 2014). This indicates that Hainan/Eksotika PSO had higher the content of unsaturated fatty acids as compared with Yanty et al.’s (2014). Hence, Hainan/Eksotika PSO had a lower melting point and higher crystallization temperature.

The extraction method can significantly affect melting behavior of PSO. SE‐PSO had higher melting points and lower crystallization temperature point than UAE‐PSO. Similarly, the extraction method and condition can affect crystallization behavior of PSO. However, the change in melting point may be ascribed to the undetectable saturated TAGs. It was found that neither solvent extraction nor aqueous enzymatic extraction made significant effects on thermal behavior of PSO (Delfanian, Esmaeilzadeh Kenari, & Sahari, 2015).

From the DSC curves of oxidations of blackberry and raspberry seed oil, three characteristic points were observed, including onset temperature (*T*_on_), the first peak (*T*_p1_), and the second peak (*T*_p2_). Among them, onset temperature (*T*_on_) and the first peak (*T*_p1_) were clearly observed in the DSC curve of the PSO oxidation. Relevant study showed when assessing the oil oxidative stability by the nonisothermal DSC method, only the onset of oxidation (*T*_on_) and the first peak (*T*_p1_) should be considered (Zhang et al., 2018). Therefore, this method was suitable for calculating activation energy and the pre‐exponential factor for the peak temperature (*T*_p1_). Table 3 shows the activation energy (*E*_a_) and the pre‐exponential factor obtained by the Kissinger method. *T*_p1_ fitted well to the experimental results for both samples (*R* ^2^ > 0.90). The *E*_a_ of UAE‐PSO (100.32 kJ/mol) was higher than that of SE‐PSO (90.51 kJ/mol), which correlated well with values reported previously for related oils, such as sunflower (90.74 kJ/mol) and sesame oils (93.55 kJ/mol), canola oil (89.94 kJ/mol), soybean oil (92.42 kJ/mol), corn oil (88.14 kJ/mol), and olive oil (86.86 kJ/mol) (Farhoosh, Niazmand, Rezaei, & Sarabi, 2008; Ghosh, Upadhyay, Mahato, & Mishra, 2019). Higher *E*_a_ values indicate a slower rate of lipid oxidation (Tan, Man, Selamat, & Yusoff, 2002); hence, UAE‐PSO proposed higher oxidative stability than SE‐PSO and most vegetable oils.

### 3.5 FTIR analysis of PSO

**Figure 2:**
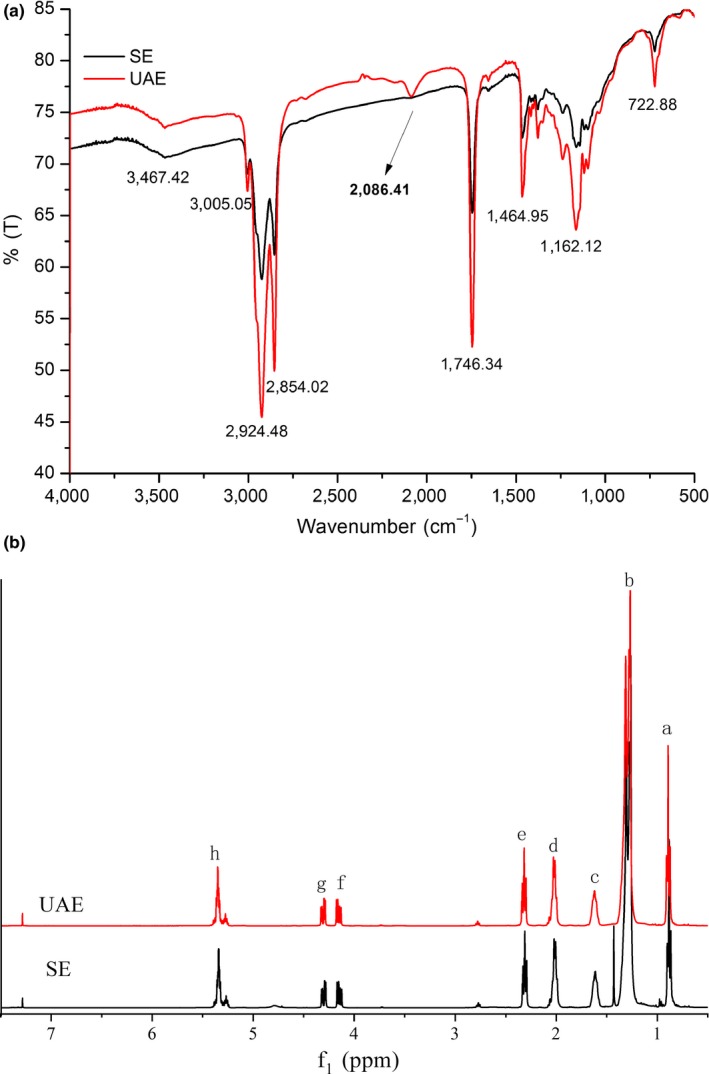
FTIR (a) and ^1^H NMR (b) spectra of papaya seed oil (PSO) from Soxhlet extraction (SE) and ultrasound‐assisted extraction (UAE)

Figure 2a shows the broad peak at 3,467/cm (O‐H stretch) and 723/cm of ortho‐substituents. The peaks at 2,924/cm and 2,853/cm were, respectively, ascribed to symmetrical and asymmetrical stretching of the C‐H in CH_2_ or CH_3_ group in fatty acids. The peak at 1,760–1,665/cm was attributed to the ester functional group. The peak at 3,000–2,700/cm was due to the carboxyl group in the free fatty acid. The peaks at 3,010–3,050/cm and 1,600–1,450/cm were due to the C=C bonds on benzene (Bhutada et al., 2016). Additionally, remarkable peak 2,086/cm (N=C=S) was observed, which indicated that the presence of benzyl was othiocyanate in UAE‐PSO. In contrast, this compound was not found in SE‐PSO.

### 3.6 ^1^HNMR analysis of PSO

The ^1^HNMR spectra of POS extracted by SE and UAE were shown in Figure 2b. The chemical shifts (*d*) and assignment of the main resonances in the ^1^HNMR spectrum were shown in Table 4. Two main regions were observed in the chemical shift regions of 6.0–4.0 and 3.0–0.5 ppm, respectively. There were totally 66 peaks detected in the ^1^H NMR spectrum. Most of the peaks were within the range of 3.0–0.5 ppm, indicating no aromatic compounds were contained in PSO. The result was basically consistent with the experimental results of Australian chia seed oil (Timilsena, Vongsvivut, Adhikari, & Adhikari, 2017). The five signals at 0.86 (a), 1.26 (b), 1.60 (c), 2.00 (d), and 2.27 (e) were assigned to terminal methyl groups, methylene protons of carbon chains, β‐carbonyl methylene protons, methylene protons of allylic groups in linoleic acid, and methylene protons in α carbonyl groups, respectively. The two duplets centered at 4.25 ppm (f) and 4.28 ppm (g) were ascribed to glyceryl methylenes. The multiplets at 5.25 ppm (h) and 5.30 ppm were ascribed to glyceryl methines and olefinic protons, respectively (Timilsena et al., 2017). After oxidation of edible oils, the oxidation products showed signals at 8.09–8.19 ppm for hydroperoxides proton and 9.30–9.90 ppm for aldehydes (Bhutada et al., 2016). Here, no hydroperoxide and aldehyde signals were found in this study, indicating that no oxidative degradation took place during SE and UAE.

**Table 4:** Chemical shifts (*d*) and assignment of the main resonances in the ^1^HNMR spectrum of papaya seed oil

δ(ppm)	Assignment	
	Proton	Compound
7.26	CHCl_3_	Chloroform (solvent)
5.30	CH=CH	All unsaturated fatty acids
5.23	CHOCOR	Triacylglycerols
4.28	CH_2_OCOR	sn‐1,3 Diacylglycerol
4.25	CH_2_OCOR	Triacylglycerols
2.77	CH=CHCH_2_CH=CH	Linoleic acid
2.27	–OCO‐CH_2_‐	Oleoyl acyl chains
2.02	CH_2_CH=CH	Linolenic acid
1.59	CH_2_CH_2_COOH	All acyl chains
1.28	(CH_2_)*_n_*	All acyl chains
1.23	(CH_2_)*_n_*	All acyl chains
0.95	CH_2_CH_2_CH_2_CH_3_	Linolenoyl
0.87		β‐sitosterol
0.85	CH=CHCH_2_CH_3_	All acids except linolenoyl

### 3.7 Analysis of microscopic changes

**Figure 3:**
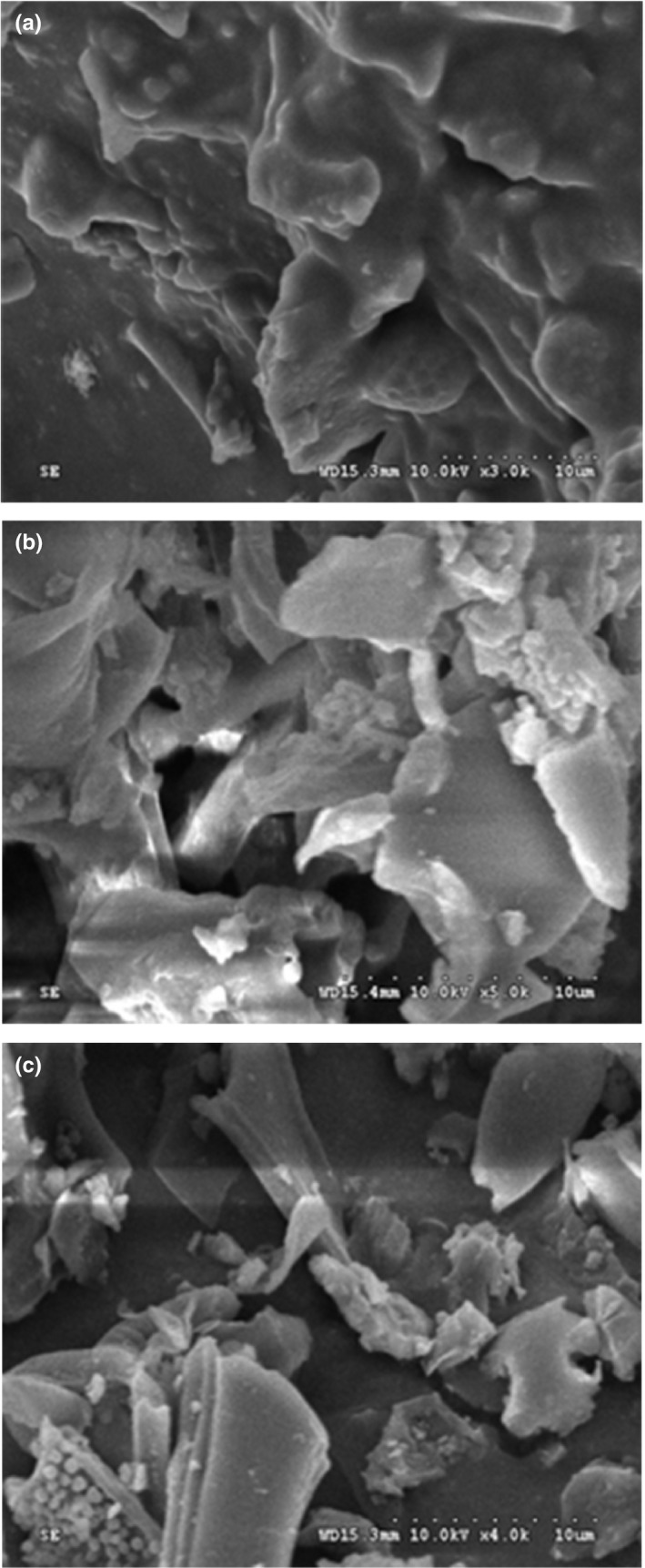
Scanning electron microscopy (SEM) of seed samples: (a) untreated seed sample; (b) after Soxhlet extraction; (c) after ultrasound‐assisted extraction under optimal conditions (1,000 × magnification)

From Figure 3a, many oil cells without signs of pitting or damage were observed on the surface of untreated seed tissues. For conventional SE (Figure 3b), more holes appeared and the structure of seed tissues became loose after removal of oil. As shown in Figure 3c, UAE slightly changed the structures of seed tissues. UAE process enhanced the diffusion of extracted solids into liquid (Hani, Torkamani, Abidin, Mahmood, & Juliano, 2017). In the process of UAE, the ultrasound power intensity might create extra vibration within sample molecules, which could cause ultrasonic cell disintegration and enhance the transformation of enzyme from solid phase to the liquid phase (Rafiquzzaman et al., 2016). The implosion of cavitation bubbles might be primary reason for the extraction enhancement. The propagation of ultrasound pressure waves produced cavitation phenomena and thus intensify extraction processes. The implosion of cavitation bubbles resulted in interparticle collisions, microturbulence, and perturbation in microporous particles, which accelerated eddy and internal diffusion. The ultrasound power damaged cell walls and membranes, which was conductive to the release of oil from the original structure. The ultrasound power intensity also enhanced the recovery of target compounds. Therefore, compared with conventional SE, UAE produced more bioactive compounds of oils.

### 3.8 Analysis of aldehyde composition of PSO

**Figure 4:**
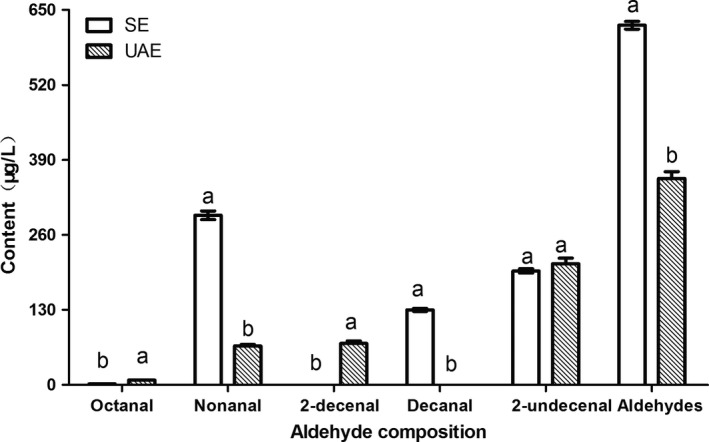
Aldehyde composition of papaya seed oil (PSO) obtained using UAE and Soxhlet extraction (SE)

As the main secondary oxidation products, aldehydes were taken as oxidation indicators. The content of oleic acid in PSO from Malaysia/Batek Batu variety, Brazil/Formosa variety, Taiwan/Tainoung variety, and Hong Kong/Sekaki variety was 73%, 71.3%, 66.74%, and 73.5%, respectively. In contrast, PSO extracted from Hainan/Eksotika variety by SBE and SCDE had lower content of oleic acid. Compared with UAE‐PSO and SE‐PSO, the content of oleic acid in PSO extracted from Hainan/Eksotika variety by UAE and SE was lower. The aldehyde composition of PSO extracted by UAE and SE was comparatively analyzed in this study. The contents of nonanal, decanal, and aldehyde in UAE‐PSO were lower than those in SE‐PSO (Figure 4). This suggested that the oxidation stability of UAE‐PSO was higher than SE‐PSO.

## 4 CONCLUSIONS

In this study, a high‐performance extraction of PSO was achieved under the optimal conditions by using UAE technology. Through analysis of physicochemical properties, it was confirmed that PSO extracted by UAE had good oil oxidative stability, with high contents of triacylglycerols and unsaturated fatty acids. The DSC analysis results showed that PSO extracted from Hainan/Eksotika had a lower melting point while a higher crystallization temperature as compared with that extracted from Batek Batu and Sekaki variety. The results of FTIR and ^1^HNMR analysis indicated the functional group existed in the extracted oil. According to SEM determination, UAE could efficiently destroy the cell walls and membranes, consequently increasing the oil release. Compared with SE‐PSO, UAE‐PSO showed higher yield and higher oxidative stability. In summary, UAE is an ideal technique to extract the PSO and other oils in food industry. PSO extracted from Hainan/Eksotika variety is expected to be processed into healthy edible oil. Further studies are needed to reveal the function and mechanism of PSO extracted from Hainan/Eksotika in vitro and in vivo.

## CONFLICT OF INTEREST

There is no conflict of interests regarding the publication of this paper.

## ETHICAL STATEMENT

This study does not involve any human or animal testing.
