# Supplementary material for: Optimized ultrasonic‐assisted extraction of papaya seed oil from Hainan/Eksotika variety
Source: Food Sci Nutr. 2019 Jul 8;7(8):2692–701. doi: 10.1002/fsn3.1125 (PMC6694413; doi:10.1002/fsn3.1125)
Supplement: Supplementary file 1 [file FSN3-7-2692-s001.docx]

**Table S1** ANOVA of the quadratic model for the oil yield during UAE process

| Source | Sum of squares^a^ | | DF^b^ | | Mean square^c^ | F-value^d^ | *p*-value^e^ | Significance^f^ |
| --- | --- | --- | --- | --- | --- | --- | --- | --- |
| Model | 95.30157 | | 9 | | 10.58906 | 6.83490 | 0.02382 | Significant |
| *X_1_* | | 56.44531 | 1 | | 56.44531 | 36.43361 | 0.00180 | Significant |
| *X_2_* | | 0.16820 | 1 | | 0.16820 | 0.10857 | 0.75514 | Not significant |
| *X_3_* | | 11.44811 | 1 | | 11.44811 | 7.38938 | 0.04186 | Significant |
| *X_1_X_2_* | | 0.02250 | 1 | | 0.02250 | 0.01452 | 0.90877 | Not significant |
| *X_1_X_3_* | | 1.01003 | 1 | | 1.01003 | 0.65194 | 0.45610 | Not significant |
| *X_2_X_3_* | | 0.03610 | | 1 | 0.03610 | 0.02330 | 0.88465 | Not significant |
| *X_1_^2^* | | 5.99054 | | 1 | 5.99054 | 3.86670 | 0.10641 | Not significant |
| *X_2_^2^* | | 0.20608 | | 1 | 0.20608 | 0.13302 | 0.73024 | Not significant |
| *X_3_^2^* | | 21.78028 | | 1 | 21.78028 | 14.05846 | 0.01330 | Significant |
| Lack of fit^g^ | | 7.35013 | | 3 | 2.45004 | 12.36770 | 0.07573 | Not significant |
| Pure error | 0.39620 | | | 2 | 0.19810 |  |  |  |
| Cor total | 103.04790 | | | 14 |  |  |  |  |
| C.V. =4.569%, *R*^2^ = 0.9248, *R*^2^_Adj_ =0.7895 | | | | | | | | |

^a^: Sum of the squared differences between the average values and the overall mean; ^b^: Degrees of freedom; ^c^:Sum of squares divided by DF; ^d^:Test for comparing term variance with residual variance; ^e^:Probability of seeing the observed F-value if the null hypothecs is true;^f^: P-value less than 0.05 indicate model term is significant; ^g^:Variation of the data around the fitted model.

Fig. S1 Response surface plots illustrating the interactive effects of different extraction parameters on extraction %yield.

**
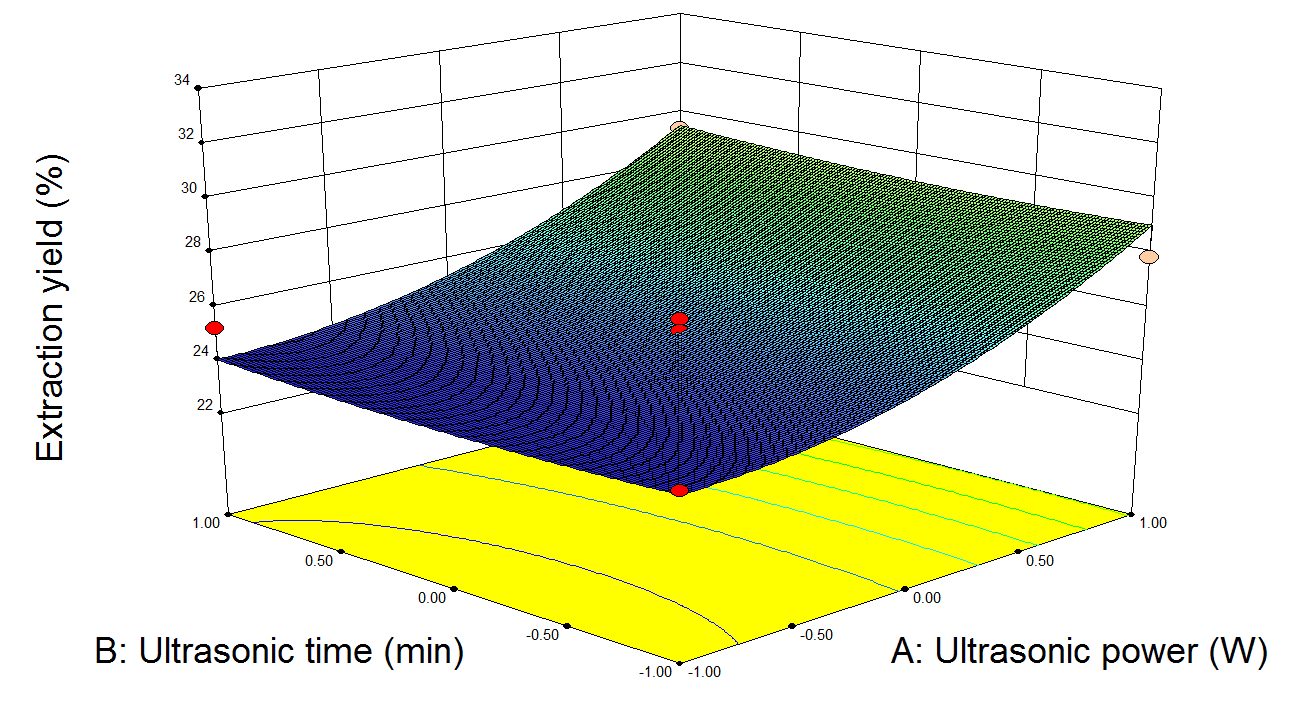
**

**
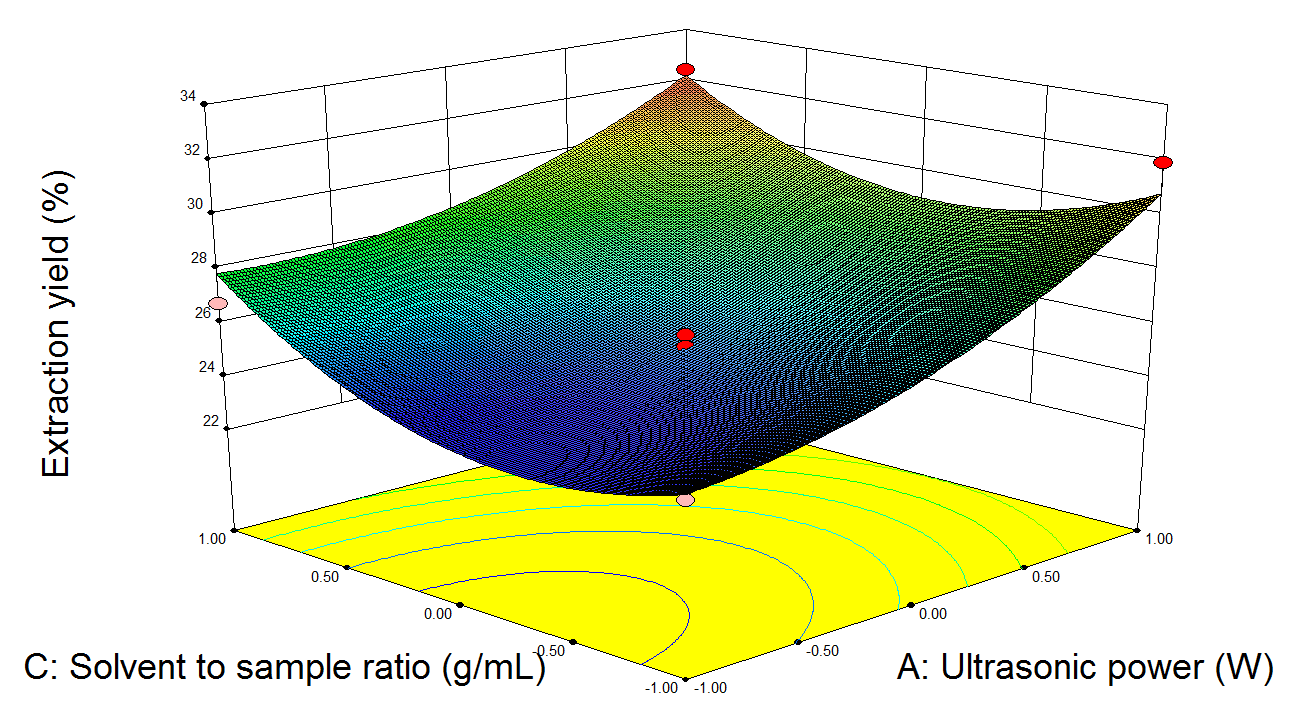
**

**
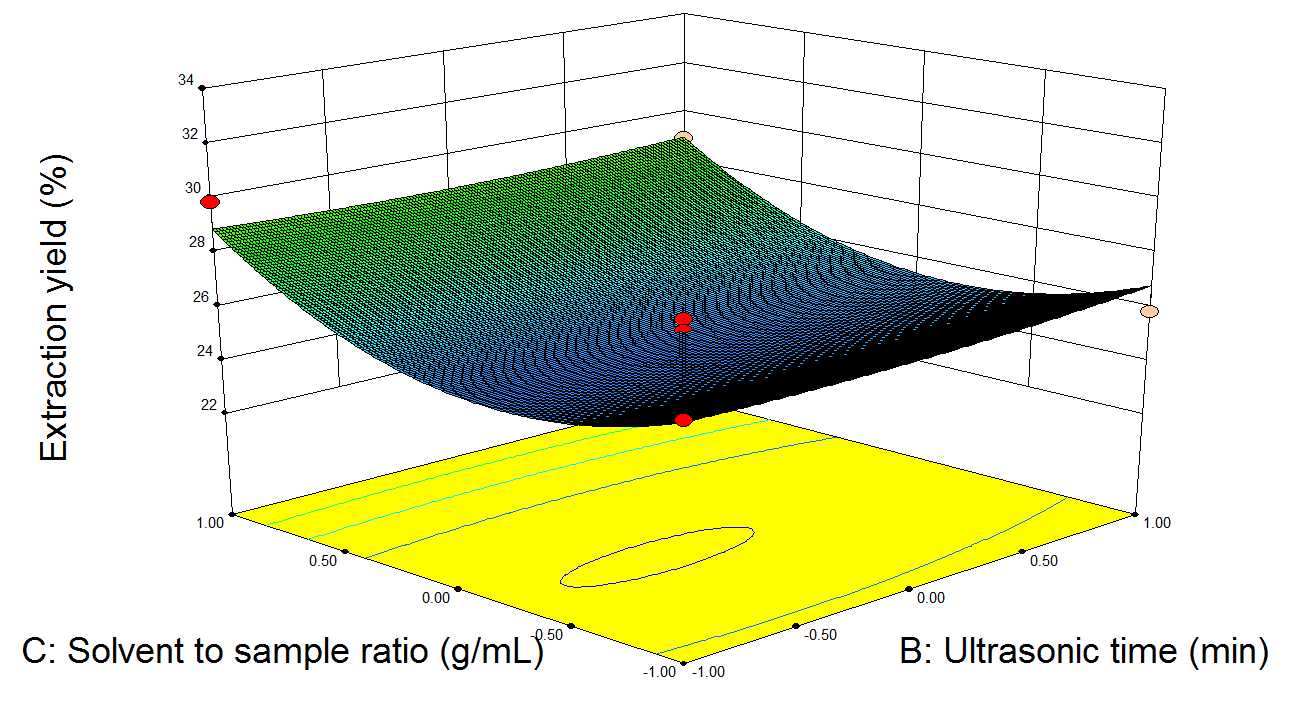
**
